# 5-Fluoro-3-(1*H*-indol-3-ylmeth­yl)-1*H*-indole

**DOI:** 10.1107/S2414314623005904

**Published:** 2023-08-04

**Authors:** Guozhe Guo

**Affiliations:** a College of Chemistry and Chemical Engineering, Longdong University, Qingyang, Gansu, 745000, People’s Republic of China; Zhejiang University (Yuquan Campus), China

**Keywords:** crystal structure

## Abstract

The title compound, C_17_H_13_FN_2_, was synthesized as a potential ligand for the construction of metal–organic frameworks. The two indole motifs present two potential coordination modes. It crystallizes in the ortho­rhom­bic system with space group *P*2_1_2_1_2_1_. The dihedral angle between the fused ring systems is 68.77 (10)°. Weak F⋯H inter­actions are observed in the crystal.

## Abstract 2

The title compound was synthesized as a potential ligand for the construction of metal–organic frameworks. The two indole motifs present two potential coordination modes. Weak F⋯H inter­actions are observed in the crystal.

## Graphical abstract

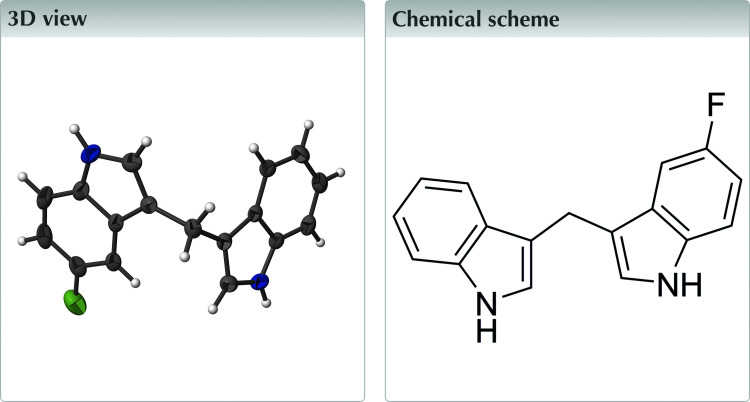

## Structure description

The di­aryl­methane motif is ubiquitous in natural products, bioactive mol­ecule and medicine (Sakurai *et al.*, 2020 ▸). The title compound belongs to the di­aryl­methane family (Safe *et al.*, 2008 ▸), which shows anti­oxidant, anti-inflammatory and anti­cancer bioactivities, and can be found in broccoli.

**Figure 1:**
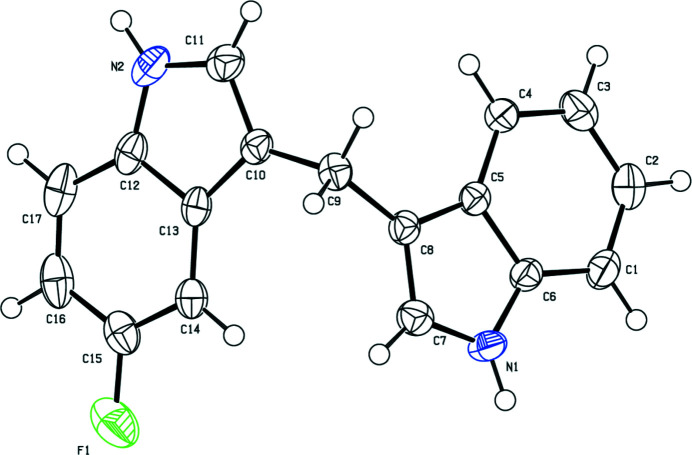
The mol­ecular structure of the title compound. Displacement ellipsoids are drawn at the 50% probability level.

**Figure 2:**
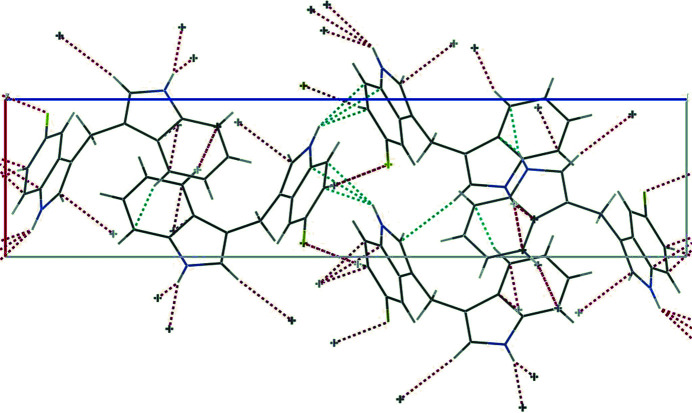
The crystal packing of the title compound. Weak inter­actions between the mol­ecules are shown as dashed lines.

In the present work, we synthesized the title compound as a potential ligand for the construction of metal–organic frameworks. This ligand is expected to be a good candidate for the construction of coordination polymers with diverse structures. The mol­ecular structure is shown in Fig. 1 ▸. The title compound crystallizes in the ortho­rhom­bic system, space group *P*2_1_2_1_2_1_. The dihedral angle between the fused ring systems is 68.77 (10)°. Fig. 2 ▸ shows the weak F⋯H and other inter­actions observed in the crystal Numerical details are given in Table 1 ▸.

## Synthesis and crystallization

To a dried reaction tube (10 ml) with a magnetic stirring bar were added NHPI (*N*-hydroxyphthalimide ester) ester (0.2 mmol), indole (0.4 mmol) and Ru(bpy)_2_(PF_6_)_2_ (3 mol %) successively. Air was then withdrawn and the tube was backfilled with argon three times. Subsequently, degassed DCM (2 ml) was injected into the tube by syringe. Then, the resulting reaction mixture was irradiated at room temperature under blue LEDs (6 W) for 12 h. The reaction progress was monitored by TLC. After the reaction was completed, the reaction mixture was concentrated under reduced pressure, and the residue was purified by column chromatography to afford the title compound. Single crystals of C_17_H_13_FN_2_ were obtained by evaporation after one week. Yield: 47 wt%.

## Refinement

Crystal data, data collection and structure refinement details are summarized in Table 2 ▸. ▸.

## Supplementary Material

Crystal structure: contains datablock(s) I. DOI: 10.1107/S2414314623005904/xu4049sup1.cif

Structure factors: contains datablock(s) I. DOI: 10.1107/S2414314623005904/xu4049Isup2.hkl

Supporting information file. DOI: 10.1107/S2414314623005904/xu4049Isup3.cml

CCDC reference: 2233347

Additional supporting information:  crystallographic information; 3D view; checkCIF report

## Figures and Tables

**Table 1:** Inter­molecular inter­actions (Å)

Atom1	Atom2	Symm. op. 2	Length	Length − vdW
F1	H16	− 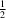 + *x*, − 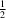 − *y*, −*z*	2.48	−0.19
C1	H4	1 − *x*, − 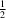 + *y*, 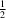 − *z*	2.88	−0.02
C4	H1	−*x*, 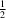 + *y*, 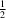 − *z*	2.55	−0.35
C5	H1	−*x*, 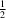 + *y*, 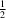 − *z*	2.58	−0.32
C11	H7	1 + *x*, *y*, *z*	2.82	−0.08
C12	H2	− 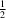 + *x*, 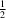 − *y*, −*z*	2.80	−0.10
C16	H2	− 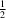 + *x*, 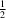 − *y*, −*z*	2.81	−0.09
C17	H2	− 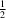 + *x*, 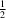 − *y*, −*z*	2.64	−0.26

**Table 2:** Experimental details

Crystal data	
Chemical formula	C_17_H_13_FN_2_
*M*_r_	264.29
Crystal system, space group	Orthorhombic, *P*2_1_2_1_2_1_
Temperature (K)	296
*a*, *b*, *c* (Å)	6.0723 (3), 7.8662 (3), 26.2693 (11)
*V* (Å^3^)	1254.78 (9)
*Z*	4
Radiation type	Mo *K*α
μ (mm^−1^)	0.09
Crystal size (mm)	0.12 × 0.1 × 0.1

Data collection	
Diffractometer	Bruker APEXII CCD
Absorption correction	Multi-scan (*SADABS*; Krause *et al.*, 2015 ▸)
*T*_min_, *T*_max_	0.691, 0.746
No. of measured, independent and observed [*I* > 2σ(*I*)] reflections	12005, 2866, 2528
*R*_int_	0.063
(sin θ/λ)_max_ (Å^−1^)	0.649

Refinement	
*R*[*F* ^2^ > 2σ(*F* ^2^)], *wR*(*F* ^2^), *S*	0.047, 0.125, 1.06
No. of reflections	2866
No. of parameters	181
H-atom treatment	H-atom parameters constrained
Δρ_max_, Δρ_min_ (e Å^−3^)	0.53, −0.30
Absolute structure	Flack *x* determined using 921 quotients [(*I* ^+^)−(*I* ^−^)]/[(*I* ^+^)+(*I* ^−^)] (Parsons *et al.*, 2013 ▸)
Absolute structure parameter	−0.1 (7)

Computer programs: *APEX2* and *SAINT* (Bruker, 2019 ▸), *SHELXT2018/2* (Sheldrick, 2015*a* ▸), *SHELXL2018/3* (Sheldrick, 2015*b* ▸) and *OLEX2* (Dolomanov *et al.*, 2009 ▸).

## full crystallographic data

### Crystal data

**Table:**

C_17_H_13_FN_2_	*D*_x_ = 1.399 Mg m^−^^3^
*M**_r_* = 264.29	Mo *K*α radiation, λ = 0.71073 Å
Orthorhombic, *P*2_1_2_1_2_1_	Cell parameters from 4629 reflections
*a* = 6.0723 (3) Å	θ = 2.7–27.4°
*b* = 7.8662 (3) Å	µ = 0.09 mm^−^^1^
*c* = 26.2693 (11) Å	*T* = 296 K
*V* = 1254.78 (9) Å^3^	Block, colourless
*Z* = 4	0.12 × 0.1 × 0.1 mm
*F*(000) = 552	

### Data collection

**Table:**

Bruker APEXII CCD diffractometer	2528 reflections with *I* > 2σ(*I*)
φ and ω scans	*R*_int_ = 0.063
Absorption correction: multi-scan (SADABS; Krause *et al.*, 2015)	θ_max_ = 27.5°, θ_min_ = 2.7°
*T*_min_ = 0.691, *T*_max_ = 0.746	*h* = −7→7
12005 measured reflections	*k* = −9→10
2866 independent reflections	*l* = −34→29

### Refinement

**Table:**

Refinement on *F*^2^	Hydrogen site location: inferred from neighbouring sites
Least-squares matrix: full	H-atom parameters constrained
*R*[*F*^2^ > 2σ(*F*^2^)] = 0.047	*w* = 1/[σ^2^(*F*_o_^2^) + (0.051*P*)^2^ + 0.4902*P*] where *P* = (*F*_o_^2^ + 2*F*_c_^2^)/3
*wR*(*F*^2^) = 0.125	(Δ/σ)_max_ < 0.001
*S* = 1.06	Δρ_max_ = 0.53 e Å^−^^3^
2866 reflections	Δρ_min_ = −0.30 e Å^−^^3^
181 parameters	Absolute structure: Flack *x* determined using 921 quotients [(*I*^+^)-(*I*^-^)]/[(*I*^+^)+(*I*^-^)] (Parsons *et al.*, 2013)
0 restraints	Absolute structure parameter: −0.1 (7)
Primary atom site location: dual	

### Special details

**Table:**

Geometry. All esds (except the esd in the dihedral angle between two l.s. planes) are estimated using the full covariance matrix. The cell esds are taken into account individually in the estimation of esds in distances, angles and torsion angles; correlations between esds in cell parameters are only used when they are defined by crystal symmetry. An approximate (isotropic) treatment of cell esds is used for estimating esds involving l.s. planes.
Refinement. The H atoms were included in calculated positions and treated as riding atoms: C–H = 0.93- 0.98 Å, with *U*_iso_(H) = 1.2Ueq (C), *U*_iso_(H) = 1.2Ueq (N).

### Fractional atomic coordinates and isotropic or equivalent isotropic displacement parameters (Å^2^)

**Table:**

	*x*	*y*	*z*	*U*_iso_*/*U*_eq_	
F1	0.0864 (4)	−0.1701 (2)	0.06215 (8)	0.0567 (6)	
N1	−0.0566 (4)	0.2923 (3)	0.23358 (10)	0.0325 (6)	
H1	−0.163457	0.235536	0.246712	0.039*	
N2	0.7008 (4)	0.3284 (3)	0.05286 (9)	0.0366 (6)	
H2	0.829218	0.336765	0.039262	0.044*	
C6	0.1230 (5)	0.3505 (3)	0.25954 (11)	0.0268 (6)	
C5	0.2620 (5)	0.4322 (3)	0.22362 (10)	0.0240 (6)	
C8	0.1535 (5)	0.4238 (3)	0.17516 (10)	0.0255 (6)	
C14	0.2106 (5)	0.1028 (3)	0.08297 (10)	0.0289 (6)	
H14	0.081609	0.123845	0.100979	0.035*	
C13	0.3747 (5)	0.2253 (3)	0.07888 (10)	0.0264 (6)	
C10	0.3965 (5)	0.3978 (3)	0.09673 (10)	0.0264 (6)	
C9	0.2277 (5)	0.4995 (3)	0.12576 (11)	0.0285 (6)	
H9A	0.288541	0.611254	0.132574	0.034*	
H9B	0.099716	0.514911	0.104165	0.034*	
C4	0.4575 (5)	0.5076 (4)	0.24071 (11)	0.0293 (6)	
H4	0.552343	0.561101	0.217991	0.035*	
C12	0.5690 (5)	0.1875 (4)	0.05142 (10)	0.0316 (6)	
C17	0.6017 (6)	0.0300 (4)	0.02794 (11)	0.0394 (8)	
H17	0.730172	0.006624	0.009970	0.047*	
C7	−0.0405 (5)	0.3383 (4)	0.18325 (11)	0.0312 (6)	
H7	−0.145343	0.315036	0.158400	0.037*	
C3	0.5069 (6)	0.5007 (4)	0.29232 (12)	0.0371 (7)	
H3	0.636564	0.549724	0.304113	0.045*	
C11	0.5953 (5)	0.4543 (4)	0.07946 (11)	0.0334 (7)	
H11	0.651263	0.562800	0.084954	0.040*	
C15	0.2462 (6)	−0.0498 (4)	0.05952 (11)	0.0345 (7)	
C1	0.1734 (6)	0.3440 (4)	0.31123 (11)	0.0344 (7)	
H1A	0.081012	0.289653	0.334300	0.041*	
C16	0.4374 (7)	−0.0887 (4)	0.03240 (12)	0.0419 (8)	
H16	0.453295	−0.195077	0.017363	0.050*	
C2	0.3651 (6)	0.4213 (4)	0.32682 (12)	0.0374 (7)	
H2A	0.401270	0.420605	0.361224	0.045*	

### Atomic displacement parameters (Å^2^)

**Table:**

	*U*^11^	*U*^22^	*U*^33^	*U*^12^	*U*^13^	*U*^23^
F1	0.0776 (17)	0.0382 (10)	0.0542 (13)	−0.0067 (11)	−0.0121 (12)	−0.0048 (9)
N1	0.0292 (14)	0.0317 (12)	0.0366 (13)	−0.0075 (10)	0.0077 (11)	0.0018 (11)
N2	0.0284 (13)	0.0539 (16)	0.0276 (12)	0.0014 (12)	0.0064 (11)	0.0075 (11)
C6	0.0278 (15)	0.0242 (12)	0.0285 (13)	−0.0002 (11)	0.0056 (12)	−0.0006 (11)
C5	0.0270 (14)	0.0189 (11)	0.0262 (13)	0.0034 (10)	0.0038 (11)	−0.0006 (10)
C8	0.0283 (14)	0.0229 (12)	0.0253 (13)	0.0017 (11)	0.0028 (11)	−0.0011 (10)
C14	0.0341 (16)	0.0307 (14)	0.0220 (13)	0.0051 (12)	−0.0021 (12)	0.0010 (10)
C13	0.0307 (15)	0.0314 (13)	0.0171 (11)	0.0064 (11)	−0.0015 (11)	0.0028 (10)
C10	0.0286 (15)	0.0310 (13)	0.0196 (12)	0.0003 (11)	−0.0006 (11)	0.0040 (10)
C9	0.0337 (15)	0.0263 (12)	0.0256 (13)	0.0023 (12)	−0.0015 (12)	0.0007 (11)
C4	0.0296 (15)	0.0283 (13)	0.0299 (14)	−0.0019 (12)	0.0038 (12)	−0.0016 (11)
C12	0.0325 (16)	0.0433 (16)	0.0191 (12)	0.0083 (13)	0.0004 (12)	0.0052 (11)
C17	0.0423 (19)	0.0527 (19)	0.0233 (14)	0.0218 (16)	0.0028 (14)	0.0028 (13)
C7	0.0293 (15)	0.0312 (14)	0.0329 (15)	−0.0005 (12)	−0.0013 (12)	−0.0012 (12)
C3	0.0363 (18)	0.0389 (16)	0.0361 (16)	−0.0004 (14)	−0.0076 (14)	−0.0065 (13)
C11	0.0344 (17)	0.0390 (16)	0.0268 (14)	−0.0031 (13)	−0.0008 (13)	0.0070 (12)
C15	0.0465 (19)	0.0304 (14)	0.0265 (14)	0.0038 (13)	−0.0086 (14)	0.0013 (11)
C1	0.0425 (18)	0.0313 (14)	0.0294 (15)	0.0008 (13)	0.0117 (13)	0.0009 (12)
C16	0.063 (2)	0.0365 (16)	0.0256 (14)	0.0183 (16)	−0.0052 (16)	−0.0024 (12)
C2	0.0442 (19)	0.0417 (16)	0.0262 (14)	0.0063 (15)	−0.0012 (14)	−0.0030 (13)

### Geometric parameters (Å, º)

**Table:**

F1—C15	1.357 (4)	C10—C9	1.507 (4)
N1—H1	0.8600	C10—C11	1.364 (4)
N1—C6	1.365 (4)	C9—H9A	0.9700
N1—C7	1.374 (4)	C9—H9B	0.9700
N2—H2	0.8600	C4—H4	0.9300
N2—C12	1.368 (4)	C4—C3	1.390 (4)
N2—C11	1.371 (4)	C12—C17	1.398 (4)
C6—C5	1.420 (4)	C17—H17	0.9300
C6—C1	1.393 (4)	C17—C16	1.372 (5)
C5—C8	1.435 (4)	C7—H7	0.9300
C5—C4	1.401 (4)	C3—H3	0.9300
C8—C9	1.497 (4)	C3—C2	1.398 (5)
C8—C7	1.373 (4)	C11—H11	0.9300
C14—H14	0.9300	C15—C16	1.396 (5)
C14—C13	1.391 (4)	C1—H1A	0.9300
C14—C15	1.366 (4)	C1—C2	1.375 (5)
C13—C10	1.442 (4)	C16—H16	0.9300
C13—C12	1.415 (4)	C2—H2A	0.9300
			
C6—N1—H1	125.2	C5—C4—H4	120.7
C6—N1—C7	109.6 (2)	C3—C4—C5	118.6 (3)
C7—N1—H1	125.2	C3—C4—H4	120.7
C12—N2—H2	125.5	N2—C12—C13	107.7 (3)
C12—N2—C11	109.0 (3)	N2—C12—C17	130.3 (3)
C11—N2—H2	125.5	C17—C12—C13	122.0 (3)
N1—C6—C5	107.1 (2)	C12—C17—H17	121.2
N1—C6—C1	130.6 (3)	C16—C17—C12	117.5 (3)
C1—C6—C5	122.3 (3)	C16—C17—H17	121.2
C6—C5—C8	107.2 (2)	N1—C7—H7	125.1
C4—C5—C6	118.8 (2)	C8—C7—N1	109.8 (3)
C4—C5—C8	133.9 (3)	C8—C7—H7	125.1
C5—C8—C9	127.7 (3)	C4—C3—H3	119.4
C7—C8—C5	106.2 (2)	C4—C3—C2	121.2 (3)
C7—C8—C9	126.0 (3)	C2—C3—H3	119.4
C13—C14—H14	121.3	N2—C11—H11	124.8
C15—C14—H14	121.3	C10—C11—N2	110.4 (3)
C15—C14—C13	117.4 (3)	C10—C11—H11	124.8
C14—C13—C10	133.9 (3)	F1—C15—C14	118.4 (3)
C14—C13—C12	119.4 (3)	F1—C15—C16	117.9 (3)
C12—C13—C10	106.7 (3)	C14—C15—C16	123.7 (3)
C13—C10—C9	127.0 (3)	C6—C1—H1A	121.3
C11—C10—C13	106.2 (3)	C2—C1—C6	117.4 (3)
C11—C10—C9	126.7 (3)	C2—C1—H1A	121.3
C8—C9—C10	115.6 (2)	C17—C16—C15	119.9 (3)
C8—C9—H9A	108.4	C17—C16—H16	120.0
C8—C9—H9B	108.4	C15—C16—H16	120.0
C10—C9—H9A	108.4	C3—C2—H2A	119.1
C10—C9—H9B	108.4	C1—C2—C3	121.7 (3)
H9A—C9—H9B	107.4	C1—C2—H2A	119.1
			
F1—C15—C16—C17	178.8 (3)	C13—C10—C11—N2	−1.1 (3)
N1—C6—C5—C8	1.5 (3)	C13—C12—C17—C16	0.0 (4)
N1—C6—C5—C4	178.5 (2)	C10—C13—C12—N2	0.3 (3)
N1—C6—C1—C2	−177.1 (3)	C10—C13—C12—C17	−179.0 (3)
N2—C12—C17—C16	−179.2 (3)	C9—C8—C7—N1	−177.8 (3)
C6—N1—C7—C8	1.6 (3)	C9—C10—C11—N2	−177.5 (3)
C6—C5—C8—C9	176.6 (3)	C4—C5—C8—C9	0.2 (5)
C6—C5—C8—C7	−0.5 (3)	C4—C5—C8—C7	−176.9 (3)
C6—C5—C4—C3	−0.6 (4)	C4—C3—C2—C1	1.2 (5)
C6—C1—C2—C3	−1.2 (5)	C12—N2—C11—C10	1.3 (3)
C5—C6—C1—C2	0.2 (4)	C12—C13—C10—C9	176.9 (3)
C5—C8—C9—C10	81.7 (4)	C12—C13—C10—C11	0.4 (3)
C5—C8—C7—N1	−0.6 (3)	C12—C17—C16—C15	0.1 (4)
C5—C4—C3—C2	−0.3 (5)	C7—N1—C6—C5	−1.9 (3)
C8—C5—C4—C3	175.5 (3)	C7—N1—C6—C1	175.8 (3)
C14—C13—C10—C9	−2.1 (5)	C7—C8—C9—C10	−101.7 (3)
C14—C13—C10—C11	−178.6 (3)	C11—N2—C12—C13	−1.0 (3)
C14—C13—C12—N2	179.5 (2)	C11—N2—C12—C17	178.3 (3)
C14—C13—C12—C17	0.2 (4)	C11—C10—C9—C8	−126.1 (3)
C14—C15—C16—C17	−0.4 (5)	C15—C14—C13—C10	178.5 (3)
C13—C14—C15—F1	−178.7 (2)	C15—C14—C13—C12	−0.4 (4)
C13—C14—C15—C16	0.6 (4)	C1—C6—C5—C8	−176.4 (3)
C13—C10—C9—C8	58.2 (4)	C1—C6—C5—C4	0.6 (4)

## References

[bb1] Bruker (2019). *APEX2* and *SAINT*. Bruker AXS Inc., Madison, Wisconsin, USA.

[bb2] Dolomanov, O. V., Bourhis, L. J., Gildea, R. J., Howard, J. A. K. & Puschmann, H. (2009). *J. Appl. Cryst.* **42**, 339–341.

[bb3] Krause, L., Herbst-Irmer, R., Sheldrick, G. M. & Stalke, D. (2015). *J. Appl. Cryst.* **48**, 3–10. 10.1107/S1600576714022985 PMC4453166 26089746

[bb4] Parsons, S., Flack, H. D. & Wagner, T. (2013). *Acta Cryst.* B**69**, 249–259. 10.1107/S2052519213010014 PMC3661305 23719469

[bb5] Safe, S., Papineni, S. & Chintharlapalli, S. (2008). *Cancer Lett.* **269**, 326–338. 10.1016/j.canlet.2008.04.021 PMC2574232 18501502

[bb6] Sakurai, S., Matsumoto, A., Kano, T. & Maruoka, K. (2020). *J. Am. Chem. Soc.* **142**, 19017–19022. 10.1021/jacs.0c09008 33017146

[bb7] Sheldrick, G. M. (2015*a*). *Acta Cryst.* A**71**, 3–8.

[bb8] Sheldrick, G. M. (2015*b*). *Acta Cryst.* C**71**, 3–8.

